# Analysis of the molecular features of rectal carcinoid tumors to identify new biomarkers that predict biological malignancy

**DOI:** 10.18632/oncotarget.4294

**Published:** 2015-06-17

**Authors:** Kei Mitsuhashi, Itaru Yamamoto, Hiroyoshi Kurihara, Shinichi Kanno, Miki Ito, Hisayoshi Igarashi, Keisuke Ishigami, Yasutaka Sukawa, Mami Tachibana, Hiroaki Takahashi, Takashi Tokino, Reo Maruyama, Hiromu Suzuki, Kohzoh Imai, Yasuhisa Shinomura, Hiroyuki Yamamoto, Katsuhiko Nosho

**Affiliations:** ^1^ Department of Gastroenterology, Rheumatology and Clinical Immunology, Sapporo Medical University School of Medicine, Sapporo, Japan; ^2^ Department of Medical Oncology, Dana-Farber Cancer Institute and Harvard Medical School, Boston, MA, USA; ^3^ Department of Gastroenterology, Keiyukai Daini Hospital, Sapporo, Japan; ^4^ Department of Medical Genome Sciences, Research Institute for Frontier Medicine, Sapporo Medical University School of Medicine, Sapporo, Japan; ^5^ Department of Molecular Biology, Sapporo Medical University School of Medicine, Sapporo, Japan; ^6^ The Institute of Medical Science, The University of Tokyo, Tokyo, Japan; ^7^ Department of Gastroenterology and Hepatology, St. Marianna University School of Medicine, Kawasaki, Japan

**Keywords:** carcinoid, non-coding RNA, epigenetics, neuroendocrine tumor, rectum

## Abstract

Although gastrointestinal carcinoid tumors are relatively rare in the digestive tract, a quarter of them are present in the rectum. In the absence of specific tumor biomarkers, lymphatic or vascular invasion is generally used to predict the risk of lymph node metastasis. We, therefore, examined the genetic and epigenetic alterations potentially associated with lymphovascular invasion among 56 patients with rectal carcinoid tumors. We also conducted a microRNA (miRNA) array analysis. Our analysis failed to detect mutations in *BRAF, KRAS, NRAS*, or *PIK3CA* or any microsatellite instability (MSI); however, we did observe CpG island methylator phenotype (CIMP) positivity in 13% (7/56) of the carcinoid tumors. The CIMP-positive status was significantly correlated with lymphovascular invasion (*P* = 0.036). The array analysis revealed that microRNA-885 (miR-885)-5p was the most up-regulated miRNA in the carcinoid tumors with lymphovascular invasion compared with that in those without invasion. In addition, high miR-885-5p expression was independently associated with lymphovascular invasion (*P* = 0.0002). In conclusion, our findings suggest that miR-885-5p and CIMP status may be useful biomarkers for predicting biological malignancy in patients with rectal carcinoid tumors.

## INTRODUCTION

Carcinoid tumors originate from neuroendocrine cells and grow slowly. Although they are relatively rare in the digestive tract, rectal carcinoid tumors account for a quarter of the gastrointestinal carcinoid tumors. Carcinoid tumors are classified as neuroendocrine tumor-grade 1 (NET-G1) tumors, according to the 2010 revision of the World Health Organization (WHO) guidelines [1]. In the absence of specific tumor biomarkers, the presence of lymphatic or vascular invasion is generally used to predict the risk of lymph node metastasis after endoscopic resection. Therefore, we examined the clinical, pathological, and molecular features of rectal carcinoid tumors to identify new biomarkers that were predictive of malignancy.

MicroRNAs (miRNAs) are a class of small non-coding RNA molecules that function as post-transcriptional gene regulators, playing a central role as master regulators of gene expression in multiple cancer-related signaling pathways, including invasion and metastasis [2–18]. Using miRNA array analysis, we recently discovered that microRNA-31 (miR-31) expression is significantly up-regulated in *BRAF*-mutated colorectal cancers compared with that in wild-type colorectal cancers [2]. Moreover, associations were identified between miR-31 expression, proximal tumor location, and poor prognosis for colorectal cancers. Nevertheless, no previous study has reported its role in the progression of rectal carcinoid tumors.

The term “the CpG island methylator phenotype (CIMP)” has been repeatedly used over the past decade to describe CpG island promoter methylation in various human malignancies [19–34]. CIMP represents a distinct form of epigenetic instability in colorectal cancer, [3, 21, 35–37] causing most sporadic colorectal cancers with microsatellite instability (MSI) through the epigenetic inactivation of *MLH1*. However, no study has reported the epigenetic alterations, including CIMP status, associated with rectal carcinoid tumors.

Therefore, we examined which molecular changes could represent new biomarkers of malignancy in rectal carcinoid tumors. To identify the associations, we analyzed the genetic and epigenetic alterations according to lymphovascular (lymphatic or vascular) invasion status using a database of 56 cases of rectal carcinoid tumors. We also conducted miRNA array analysis to detect the miRNA molecules that are potentially associated with lymphovascular invasion.

## RESULTS

### Genetic and epigenetic alterations in rectal carcinoid tumors

**Figure 1:**
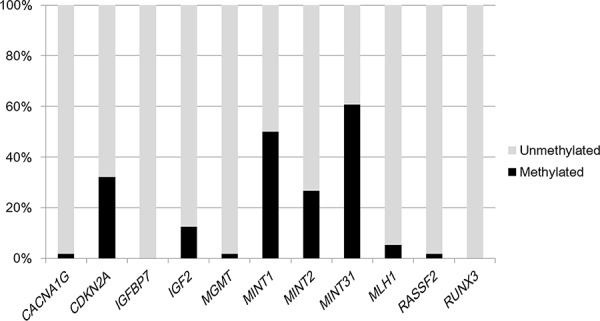
*Frequencies of CACNA1G, CDKN2A* (*p16*), *IGFBP7, IGF2, MGMT, MINT1, MINT2, MINT31, MLH1, RASSF2*, and *RUNX3* methylations in 56 rectal carcinoid tumors

None of the *BRAF, KRAS, NRAS*, or *PIK3CA* mutations were detected in the carcinoid tumors; moreover, none of the 56 cases exhibited MSI-high status. With regard to the epigenetic alterations, *CACNA1G, CDKN2A* (*p16*), *IGFBP7, IGF2, MGMT, MINT1, MINT2, MINT31, MLH1, RASSF2*, and *RUNX3* methylations were sequentially detected in 1 (1.8%), 18 (32%), 0 (0%), 7 (13%), 1 (1.8%), 28 (50%), 15 (27%), 34 (61%), 3 (5.4%), 1 (1.8%), and 0 (0%) rectal carcinoid tumors, respectively (Figure 1).

### The association between lymphovascular invasion and clinical and molecular features in rectal carcinoid tumors

Table 1 shows the association between lymphovascular invasion and clinical and molecular features in carcinoid tumors. Neither age nor gender was correlated with lymphovascular invasion. With regard to the molecular features, *IGF2* (*P* = 0.036), *MINT31* (*P* = 0.034), and *MLH1* (*P* = 0.0022) methylations were significantly correlated with lymphovascular invasion.

**Table 1:** Clinical and molecular features of rectal carcinoid tumors by lymphovascular invasion

Clinical and molecular feature	Total	Lymphovascular invasion		*P*
		Negative	Positive	
All cases	56	43	13	
Gender				
Male	29 (52%)	21 (49%)	8 (62%)	0.42
Female	27 (48%)	22 (51%)	5 (38%)	
Age (mean ± SD)	58.9 ± 13.9	58.3 ± 2.1	61.0 ± 3.8	0.53
Tumor size (mm) (mean ± SD)	6.2 ± 2.5	5.9 ± 0.37	7.0 ± 0.68	0.17
*BRAF* (codon 600)				
Wild-type	56 (100%)	43 (100%)	13 (0%)	N/A
Mutated	0 (0%)	0 (0%)	0 (0%)	
*KRAS* (codon12, 13, 61, or 146)				
Wild-type	56 (100%)	43 (100%)	13 (0%)	N/A
Mutated	0 (0%)	0 (0%)	0 (0%)	
*NRAS* (codon 12, 13, or 61)				
Wild-type	56 (100%)	43 (100%)	13 (0%)	N/A
Mutated	0 (0%)	0 (0%)	0 (0%)	
*PIK3CA* (exon 9 or 20)				
Wild-type	56 (100%)	43 (100%)	13 (0%)	N/A
Mutated	0 (0%)	0 (0%)	0 (0%)	
*CACNA1G*				
Unmethylated	55 (98%)	42 (98%)	13 (100%)	0.47
Methylated	1 (1.8%)	1 (2.3%)	0 (0%)	
*CDKN2A* (*p16*)				
Unmethylated	38 (68%)	32 (74%)	6 (46%)	0.062
Methylated	18 (32%)	11 (26%)	7 (54%)	
*IGFBP7*				
Unmethylated	56 (100%)	43 (100%)	13 (0%)	N/A
Methylated	0 (0%)	0 (0%)	0 (0%)	
*IGF2*				
Unmethylated	49 (87%)	40 (93%)	9 (69%)	0.036
Methylated	7 (13%)	3 (7.0%)	4 (31%)	
*MGMT*				
Unmethylated	55 (98%)	43 (100%)	12 (92%)	0.084
Methylated	1 (1.8%)	0 (0%)	1 (7.7%)	
*MINT1*				
Unmethylated	28 (50%)	22 (51%)	6 (46%)	0.75
Methylated	28 (50%)	21 (49%)	7 (54%)	
*MINT2*				
Unmethylated	41 (73%)	33 (77%)	8 (62%)	0.29
Methylated	15 (27%)	10 (23%)	5 (38%)	
*MINT31*				
Unmethylated	22 (39%)	20 (47%)	2 (15%)	0.034
Methylated	34 (61%)	23 (53%)	11 (85%)	
*MLH1*				
Unmethylated	53 (95%)	43 (100%)	10 (77%)	0.0022
Methylated	3 (5.4%)	0 (0%)	3 (23%)	
*RASSF2*				
Unmethylated	55 (98%)	43 (100%)	12 (92%)	0.084
Methylated	1 (1.8%)	0 (0%)	1 (7.7%)	
*RUNX3*				
Unmethylated	56 (100%)	43 (100%)	13 (100%)	N/A
Methylated	0 (0%)	0 (0%)	0 (0%)	
CIMP status				
CIMP negative	49 (87%)	40 (93%)	9 (69%)	0.036
CIMP positive	7 (13%)	3 (7%)	4 (31%)	
MSI status				
MSI-low/MSS	56 (100%)	43 (100%)	13 (100%)	N/A
MSI-high	0 (0%)	0 (0%)	0 (0%)	

Percentages (%) indicate the proportion of cases with a specific clinical or molecular feature within a given dichotomous category of lymphovascular invasion status. CIMP positivity was defined as the presence of four or more of the six methylated promoters [*CDKN2A* (*p16*), *IGF2, MINT1, MINT2, MINT31*, and *MLH1*]. The *P*-values were calculated using *t*-test for age and tumor size and by means of the χ^2^ test or Fisher's exact test for all other variables.

CIMP, CpG island methylator phenotype; MSI, microsatellite instability; N/A, not applicable; SD, standard deviation.

### The association between lymphovascular invasion and CIMP status in rectal carcinoid tumors

We selected the six markers [*CDKN2A* (*p16*), *IGF2, MLH1, MINT1, MINT2*, and *MINT31*] that were methylated in more than 5% of the rectal carcinoid tumors as CIMP markers. CIMP-positive status was defined as the presence of four or more of the six methylated promoters and CIMP-negative status as zero to three of the six methylated promoters. The CIMP-positive cases were detected in 13% (8/56) of the rectal carcinoid tumors, and the CIMP status was significantly correlated with lymphovascular invasion (*P* = 0.036) (Table 1).

### Detection of high microRNA-885-5p expression in rectal carcinoid tumors with lymphovascular invasion on miRNA array analysis

To examine the miRNA expression signature in rectal carcinoid tumors with lymphovascular invasion, 10 carcinoid tumors were randomly selected from the carcinoid tumor specimens for miRNA array analysis. Lymphovascular invasion was positive in five cases and negative in five. The median expression levels in the carcinoid tumors with lymphovascular invasion were compared with those in the tumors without invasion. The miRNA array data revealed different expression levels in individual miRNAs between the two groups. Ten miRNAs that displayed higher expression levels (≥ 30-fold change) in carcinoid tumors with lymphovascular invasion than those without invasion are shown in Table 2. Of the 760 miRNAs, microRNA-885 (miR-885)-5p was the most up-regulated in the carcinoid tumors with lymphovascular invasion compared with that in those without invasion (878-fold change, *P* = 0.037).

**Table 2:** Differentially expressed microRNAs in lymphovascular invasion positive and negative rectal carcinoid tumors by microRNA array analysis

No.	Name of microRNA(miR Base ID)	Relative microRNA expression (microRNA/U6)			*P*
		Lymphovascular invasion		Fold change (Positive group / Negative group)	
		Negative group (median; *N* = 5)	Positive group (median; *N* = 5)		
1	hsa-miR-885-5p	0.053	46.6	878	0.037
2	hsa-miR-216b	0.032	5.9	187	0.32
3	hsa-miR-204	0.27	43.6	160	0.057
4	hsa-miR-198	1.3	159.5	122	0.17
5	hsa-miR-135a	6.0	582.0	96	0.049
6	hsa-miR-452	0.39	15.9	41	0.79
7	hsa-miR-216a	0.0012	0.047	38	0.15
8	hsa-miR-486-3p	0.39	14.6	38	0.024
9	mmu-miR-499	0.36	13.4	38	0.056
10	hsa-miR-146b-3p	0.44	14.6	33	0.083

The fold change is expressed as the median of that for the rectal carcinoid tumors with lymphovascular invasion divided by those without lymphovascular invasion for each microRNA. *P*-values were determined by the Mann-Whitney *U* test.

### Distribution of miR-885-5p expression in rectal carcinoid tumors and its association with clinical and molecular features

We assessed all cases of 56 rectal carcinoid tumors based on the availability of miR-885-5p expression levels quantified in the carcinoid tumor and paired normal mucosa specimens. The expression of miR-885-5p was calculated using the equation 2^−ΔCT^, where ΔC_T_ = (C_T_ miR-885 − C_T_ U6). To calculate the relative expression of miR-885-5p in each carcinoid tumor, 2^−ΔCT^ of the tumor tissue was divided by 2^−ΔCT^ of paired normal tissue. The distributions of miR-885-5p expression across the 56 tumor specimens were as follows: mean, 135.0; median, 67.1; standard deviation (SD), 148.5; range, 4.3–538.2; interquartile range, 20.6–188.9. The cases with miR-885-5p expression were then divided into quartiles for further analysis: Q1 (< 20.6), Q2 (20.6–67.0), Q3 (67.1–188.8), and Q4 (≥ 188.9). Table 3 shows the clinical features of the carcinoid tumors according to their miR-885-5p expression level. High miR-885-5p expression was significantly correlated with lymphovascular invasion (*P* < 0.0001) but not with gender, age, or tumor size. With regard to epigenetic alterations, *MLH1* methylation was only significantly associated with miR-885-5p expression (*P* = 0.0090).

**Table 3:** Clinicopathological and molecular features of rectal carcinoid tumors by miR-885-5p expression

Clinicopathological and molecular feature	Total	miR-885-5p		*P*
		Low expression	High expression	
All cases	56	51	5	
Gender				
Male	29 (52%)	26 (51%)	3 (60%)	0.70
Female	27 (48%)	25 (49%)	2 (40%)	
Age (mean ± SD)	58.9 ± 13.9	57.2 ± 6.2	59.1 ± 1.9	0.78
Tumor size (mm) (mean ± SD)	6.2 ± 2.5	7.0 ± 1.1	6.1 ± 0.35	0.44
Lymphovascular invasion				
Negative	43 (77%)	43 (84%)	0 (0%)	<0.0001
Positive	13 (23%)	8 (16%)	5 (100%)	
*CDKN2A* (*p16*)				
Unmethylated	38 (68%)	36 (71%)	2 (40%)	0.18
Methylated	18 (32%)	15 (29%)	3 (60%)	
*IGF2*				
Unmethylated	49 (87%)	46 (90%)	3 (60%)	0.097
Methylated	7 (13%)	5 (9.8%)	2 (40%)	
*MINT1*				
Unmethylated	28 (50%)	25 (49%)	3 (60%)	0.64
Methylated	28 (50%)	26 (51%)	2 (40%)	
*MINT2*				
Unmethylated	41 (73%)	38 (75%)	3 (60%)	0.50
Methylated	15 (27%)	13 (25%)	2 (40%)	
*MINT31*				
Unmethylated	22 (39%)	20 (39%)	2 (40%)	0.97
Methylated	34 (61%)	31 (61%)	3 (60%)	
*MLH1*				
Unmethylated	53 (95%)	50 (98%)	3 (60%)	0.0090
Methylated	3 (5.3%)	1 (2.0%)	2 (40%)	
CIMP status				
Negative	7 (13%)	5 (9.8%)	2 (40%)	0.098
Positive	49 (87%)	46 (90%)	3 (60%)	

Percentage (%) indicates the proportion of cases with a specific clinicopathological or molecular feature according to miR-885-5p expression status. CIMP positivity was defined as the presence of four or more of the six methylated promoters [*CDKN2A* (*p16*), *IGF2, MINT1, MINT2, MINT31*, and *MLH1*]. *P*-values were calculated by *t*-test for age and tumor size and by a chi-square test or Fisher's exact test for all other variables.

CIMP, CpG island methylator phenotype; miR-885, microRNA-885; SD, standard deviation.

### Multivariate analysis to identify the association with lymphovascular invasion in carcinoid tumors

To account for multiple hypothesis testing in associations between the lymphovascular invasion status and the other five covariates (gender, age, tumor size, CIMP status, and miR-885-5p), the *P*-value was adjusted by Bonferroni correction to *P* = 0.01 (= 0.05/5). A backward stepwise elimination with a threshold of *P* = 0.20 was used to select variables in the final model. Of the variables, only CIMP status and miR-885-5p were included in the final model, which showed that lymphovascular invasion was only significantly associated with high miR-885-5p expression (*P* = 0.0002).

## DISCUSSION

We examined the genetic and epigenetic alterations associated with lymphovascular invasion and conducted miRNA array analysis among 56 patients with rectal carcinoid tumors and who underwent endoscopic resection. We did not detect *KRAS, BRAF*, or *PIK3CA* mutations or MSI-high in the carcinoid tumors. With regard to the epigenetic alterations, CIMP positivity was present in 13% (7/56) of the rectal carcinoid tumors and was significantly correlated with lymphovascular invasion. The miRNA array analysis revealed that miR-885-5p was the most up-regulated miRNA in the rectal carcinoid tumors with lymphovascular invasion compared with that in those without invasion. Our data also showed that high miR-885-5p expression was independently associated with lymphovascular invasion.

Given that miRNAs can function as oncogenes or tumor suppressors, they are increasingly being recognized as useful biomarkers for various human cancers [13–18]. In colorectal cancers, several miRNAs are known to be deregulated [2–12, 38–40] with target genes in the downstream effectors of epidermal growth factor receptor (EGFR) [2, 9, 41, 42]. With regard to premalignant colorectal lesions, we recently noted that high miR-31 expression is frequently detected in cases with serrated lesions [sessile serrated adenoma/polyp (SSA/P) and traditional serrated adenoma] than in non-serrated adenomas, suggesting an association between miR-31 expression and the serrated pathway [3].

In this study, specific miRNA expression associated with lymphovascular invasion was identified in the rectal carcinoid tumors. Using miRNA array analysis, our data showed that miR-885-5p expression was the most up-regulated miRNA in the carcinoid tumors with lymphovascular invasion compared with that in those without invasion (878-fold change). In the current study, we found that high miR-885-5p expression was independently associated with lymphovascular invasion in a sample of 56 patients with rectal carcinoid tumors. To our knowledge, this is therefore the first report that has identified an association between miR-885-5p expression and lymphovascular invasion in rectal carcinoid tumors.

MiR-885-5p is located in the 3p25.3 genomic region. A previous study reported that the up-regulation of miR-885-5p reduced the levels of MMP-9 in glioma cells and that it inhibited cellular invasion [43]. Furthermore, the transfection of miR 885-5p mimics could decrease MMP-9 expression. Because MMP-9 is known to play a pivotal role in regulating invasiveness in various human cancers, miR-885-5p may increase for inhibiting tumor cell invasion via MMP-9 in rectal carcinoid tumors. Our current study was limited by the cross-sectional design and the potential for bias (i.e., selection bias) that could have confounded the results. Nevertheless, our multivariate regression analysis was adjusted for potential clinical and molecular confounders. Although further study is needed to confirm our results, this is an interesting first step toward the improved understanding and diagnosis of these rare tumors.

With regard to colorectal cancer, a recent miRNA array analysis reported that miR-885-5p is significantly up-regulated in liver metastases compared with that in primary colorectal cancer tissue, and high serum miR-885-5p expression significantly predicts survival and metastasis in patients with colorectal cancer [44]. Although miR-885-5p expression in primary colorectal cancer tissue was not previously associated with clinical and pathological features, high serum miR-885-5p is significantly associated with both poor overall survival and poor disease-free survival [44]. Using *in situ* hybridization analysis, Hur et al. have also confirmed miR-885-5p expression in metastatic tumor cells of the liver, but not in their adjacent hepatocytes; however, they did not utilize laser capture microdissection, which might decrease the influence of stromal cells [44]. When Iino et al. used laser capture microdissection to isolate cancer cells from formalin-fixed paraffin-embedded (FFPE) primary colorectal cancer samples and corresponding metastatic liver tumors, they identified miR-885-5p as the second most up-regulated miRNA in colorectal liver metastases compared with primary tumors [45]. These findings suggest that the up-regulation of miR-885-5p in tumor cells, but not in stromal cells, may play an important role in the development of colorectal liver metastasis.

CIMP is associated with both favorable and unfavorable prognoses, as well as different clinical characteristics, depending on the tumor type. In colorectal cancer, CIMP is associated with a favorable prognosis [35, 46]. In contrast, a recent analysis in patients with primary clear cell renal carcinoma showed that CIMP positivity is characterized by tumor clusters that are associated with aggressiveness and patient survival [34]. Regarding early colorectal neoplastic lesions, we recently reported that there was a progressive increase in the methylation status (i.e., CIMP status and *MLH1* methylation) from hyperplastic polyps to SSA/Ps to SSA/Ps with cytological dysplasia [3], suggesting that accumulating epigenetic alterations may be associated with biological malignancy in serrated pathway progression. Such findings emphasize the motivation for establishing whether CIMP is universal or cancer specific because of its potential for use as a prognostic marker. There are several possible explanations for the discrepancies identified to date. First, although CIMP has been identified in different tumors, it may simply not be a universal marker of good or bad prognosis. Second, it is possible that the gene panels and cut-off thresholds used to define CIMP are not sufficiently accurate to define the true phenotypes of some cancers.

In contrast to colorectal cancer, research into CIMP in rectal carcinoid tumors is unfamiliar. In the current study, we examined 11 methylated promoters. CIMP positivity was defined as the presence of four or more of the six methylated promoters [*CDKN2A* (*p16*), *IGF2, MINT1, MINT2, MINT31*, and *MLH1*], which was detected in 13% of the rectal carcinoid tumors and was associated with lymphovascular invasion. These results suggest that some epigenetic alterations were associated with biological malignancy in rectal carcinoid tumors.

The *BRAF, KRAS*, and *NRAS* mutations are widely recognized to be the major causes of RAS/RAF/MEK/ERK pathway dysregulation in colorectal cancer [2, 9, 41, 42]. Previous studies reported that *BRAF, KRAS*, and *NRAS* mutations occur in 10%–15% [2, 35, 36, 47], 35–40% [36, 37, 42, 48], and 2–7% of colorectal cancers [42, 49], respectively. The *PIK3CA* gene encodes the catalytic subunit p110 alpha of PI3K. Mutant *PIK3CA* stimulates the PI3K/AKT pathway and promotes cell growth in various cancers, including colorectal cancer. *PIK3CA* (exon 9 and 20) mutations have been described in 15–18% of colorectal cancers [47, 50]. In the current study, we found no evidence of the *BRAF, KRAS, NRAS*, or *PIK3CA* mutations in rectal carcinoid tumors, suggesting that neither the RAS/RAF/MEK/ERK pathway nor the PI3K/AKT pathway were associated with tumorigenesis and disease progression in those tumors.

In conclusion, we demonstrated that CIMP positivity was significantly correlated with lymphovascular invasion in rectal carcinoid tumors. Furthermore, miR-885-5p was independently associated with lymphovascular invasion in carcinoid tumors. Thus, our findings suggest that miR-885-5p and CIMP status may be useful biomarkers for predicting biological malignancy in patients with rectal carcinoid tumors.

## MATERIALS AND METHODS

### Patients and rectal carcinoid tumor tissue specimens

We collected formalin-fixed paraffin-embedded (FFPE) tissue specimens from 56 cases of rectal carcinoid tumors following endoscopic resection at Sapporo Medical University Hospital or Keiyukai Sapporo Hospital between 2007 and 2014. To minimize selection bias, we collected consecutive FFPE tissue specimens. All lesions were submucosal with smooth surfaces and yellow tones, pathologically corresponding to the WHO NET-G1 classification [1].

Lymphovascular (lymphatic or vascular) invasion was detected in 23% (13/56) of the rectal carcinoid tumors. Of the 13 cases with lymphovascular invasion, surgical resections were treated in 7 cases. Several cases (*N* = 6) were followed without surgical resection because informed consent was not obtained from those patients. Lymphovascular invasion was used as a surrogate marker of the malignancy potential. No patient exhibited recurrence or death associated with carcinoid tumor until December 2014.

Informed consent was obtained from all the patients before specimen collection. This study was approved by the institutional review boards of the participating institutions and complied with the tenets of the Helsinki Declaration. Our analysis of the rectal carcinoid tumor tissue specimens is fully compliant with the REMARK guidelines [51].

### Pyrosequencing of *BRAF, KRAS, NRAS*, and *PIK3CA* and analysis of MSI

Genomic DNA was used for PCR and targeted pyrosequencing of *BRAF* (codon 600), *KRAS* (codon 12, 13, 61, or 146), *NRAS* (codon 12, 13, or 61), and *PIK3CA* (exon 9 or 20) as previously described [2, 42, 47, 49, 50, 52]. MSI analysis was performed using two markers (BAT25 and BAT26) as previously described [2, 48].

### Sodium bisulfite treatment and Real-Time PCR (MethyLight) to assess promoter methylation of *CACNA1G, CDKN2A* (*p16*), *IGFBP7, IGF2, MGMT, MINT1, MINT2, MINT31, MLH1, RASSF2*, and *RUNX3*

Bisulfite modification of genomic DNA was performed using the BisulFlash™ DNA Modification Kit (Epigentek, Brooklyn, NY, USA) [3]. DNA methylation was quantified in 11 promoters [*CACNA1G, CDKN2A* (*p16*), *IGFBP7, IGF2, MGMT, MINT1, MINT2, MINT31, MLH1, RASSF2*, and *RUNX3*] using Real-Time PCR (MethyLight) as previously described [35–37, 48]. The percentage of methylated reference (i.e., methylation index) at a specific locus was calculated as previously described [35, 36, 53]. Methylation positivity was defined as the percentage of methylated reference ≥ 4 as previously validated [35, 36, 53].

### RNA extraction and microRNA array analysis

Total RNA was extracted from FFPE tissues using the miRNeasy FFPE Kit (Qiagen, Valencia, CA, USA). The TaqMan^®^ Array Human MicroRNA A + B Cards Set v3.0 (Applied Biosystems, Foster City, CA, USA) was used for simultaneous measurement of the expression of 760 miRNAs on a microfluidic PCR platform. In brief, 1 μg of total RNA was reverse transcribed using the Megaplex Pools Kit (Applied Biosystems), following which miRNAs were amplified and detected by PCR with specific primers and TaqMan probes. PCR was run in the 7900HT Fast Real-Time PCR system (Applied Biosystems), and SDS 2.2.2 software (Applied Biosystems) was used for comparative analysis of the cycle threshold (ΔC_T_). U6 snRNA (RNU6B; Applied Biosystems) served as an endogenous control. ΔC_T_ was calculated by subtracting the C_T_ values of U6 from the C_T_ values of the gene of interest. Expression of each miRNA in the tumor samples was calculated using the equation 2^−ΔCT^, where ΔC_T_ = (C_T_ miRNA − C_T_ U6).

### Quantitative reverse transcription-PCR (RT-PCR) of microRNA-885-5p

MiR-885-5p expression levels were analyzed by quantitative RT-PCR using the TaqMan MicroRNA Reverse Transcription Kit (Applied Biosystems) and TaqMan microRNA Assays (Applied Biosystems) as previously described [2]. U6 small nuclear RNA (snRNA; RNU6B; Applied Biosystems) served as an endogenous control. We defined high expression level groups of miR-885-5p as the fourth level (Q4) in a quartile as described previously [2].

### Statistical analysis

The JMP (version 10) software was used for all statistical analyses (SAS Institute, Cary, NC, USA). *P* values were two-sided. Univariate analysis was performed to determine the clinical and molecular characteristics according to the lymphovascular invasion of the rectal carcinoid tumors. *P* values were calculated using *t*-test for age and tumor size and by a chi-square test or Fisher's exact test for all other variables.

A multivariate logistic regression analysis assessing the relationships with lymphovascular invasion status initially included gender (male vs. female), age (continuous), tumor size (continuous), CIMP status (positive vs. negative) and miR-885-5p expression (high expression vs. low expression), considering potential confounding and causal relationships.

## Abbreviations

CIMP: CpG island methylator phenotype
EGFR: epidermal growth factor receptor
FFPE: formalin-fixed paraffin-embedded
miRNA: microRNA
miR-31: microRNA-31
miR-885: microRNA-885
MSI: microsatellite instability
NET-G1: neuroendocrine tumor-grade 1
RT-PCR: reverse transcription-PCR
SD: standard deviation
SSA/P: sessile serrated adenoma/polyp
WHO: World Health Organization
